# Sensory Abnormalities in Focal Hand Dystonia and Non-Invasive Brain Stimulation

**DOI:** 10.3389/fnhum.2014.00956

**Published:** 2014-12-05

**Authors:** Angelo Quartarone, Vincenzo Rizzo, Carmen Terranova, Demetrio Milardi, Daniele Bruschetta, Maria Felice Ghilardi, Paolo Girlanda

**Affiliations:** ^1^Department of Neurosciences, Psychiatry and Anaesthesiological Sciences, University of Messina, Messina, Italy; ^2^Department of Physiology, Pharmacology and Neuroscience, City University of New York (CUNY) Medical School, New York, NY, USA; ^3^IRCCS Centro Neurolesi Bonino-Pulejo, Messina, Italy; ^4^Department of Biomedical Science and Morphological and Functional Images, University of Messina, Messina, Italy

**Keywords:** sensory-motor integration, transcranial magnetic stimulation, cortical plasticity, LTP, rehabilitation, focal dystonia

## Abstract

It has been proposed that synchronous and convergent afferent input arising from repetitive motor tasks may play an important role in driving the maladaptive cortical plasticity seen in focal hand dystonia (FHD). This hypothesis receives support from several sources. First, it has been reported that in subjects with FHD, paired associative stimulation produces an abnormal increase in corticospinal excitability, which was not confined to stimulated muscles. These findings provide support for the role of excessive plasticity in FHD. Second, the genetic contribution to the dystonias is increasingly recognized indicating that repetitive, stereotyped afferent inputs may lead to late-onset dystonia, such as FHD, more rapidly in genetically susceptible individuals. It can be postulated, according to the two factor hypothesis that dystonia is triggered and maintained by the concurrence of environmental factors such as repetitive training and subtle abnormal mechanisms of plasticity within somatosensory loop. In the present review, we examine the contribution of sensory-motor integration in the pathophysiology of primary dystonia. In addition, we will discuss the role of non-invasive brain stimulation as therapeutic approach in FHD.

## Introduction

Task-specific focal dystonia is characterized by excessive and inappropriate muscle activation during highly skilled fine motor tasks, resulting in slow, clumsy movements, and impaired task performance. The condition typically affects highly trained, stereotypical movement patterns, such as writing (writer’s cramp) or playing a musical instrument (musician’s dystonia) (Byl, 2003; Candia et al., 2003). Despite dystonia is recognized as a motor disorder, it is not uncommon for patients with focal dystonia to complain of mild sensory symptoms such as pain or discomfort in the affected area before dystonic symptoms develop (Ghika et al., 1993; Martino et al., 2005), while conventional clinical sensory examination is usually normal. Indeed, there are numerous research reports documenting that patients with focal dystonia have an impaired ability in discriminating tactile stimuli in both the spatial and temporal domain (Tinazzi et al., 1999; Bara-Jimenez et al., 2000; Scontrini et al., 2009). Abnormalities in the temporal discrimination have been shown in unaffected carriers of the DYT1 mutation suggesting that such abnormality may precede the overt manifestations of dystonia (Fiorio et al., 2007). Moreover, proprioceptive-based finger position sense thresholds and the perception of arm motion have been reported to be abnormal in patients with cervical dystonia or blepharospasm (Grünewald et al., 1997; Putzki et al., 2006). The abnormalities in both tactile and proprioceptive processing are not restricted to the affected dystonic musculature, but were also documented in non-affected body regions (Molloy et al., 2003; Putzki et al., 2006; Fiorio et al., 2008). Altogether these findings indicate that a generalized somatosensory deficit in focal dystonia where the abnormalities of detecting and discriminating somatosensory stimuli represent a widespread endophenotypic trait. We hypothesize that dystonia is triggered and maintained by the presence of environmental factors and subtle plasticity abnormalities within somatosensory loop.

In the present paper, we would like to review sensory abnormalities in dystonia and to explore how these informations can be exploited for new therapeutic strategies employing non-invasive brain stimulation (NIBS).

## Abnormal Sensory-Motor Integration in Dystonia

The integration of inhibitory, mainly proprioceptive inputs from adjacent body parts is impaired in dystonia both at cortical and spinal level. Reciprocal inhibition is the central nervous system process in which a muscle is inhibited when its antagonist is activated. It has several components and the second longer phase of reciprocal inhibition, tested at the spinal level, is absent in patients with focal hand dystonia (FHD) (Nakashima et al., 1989). It has been proposed that defective inhibitory mechanisms within the circuits of reciprocal inhibition might underlie co-contraction. The inhibitory interactions between antagonist muscles are also abnormal at the sensory-motor cortex level (Bertolasi et al., 2003). Interestingly, intramuscular injection of botulinum toxin in dystonic patients can successfully ameliorate the involuntary muscle activity and improve the reciprocal inhibition by increasing presynaptic inhibition (Priori et al., 1995). Sensorimotor processing of muscle spindle afferent discharges has been found to be abnormal in dystonic patients (Hallett, 1995). Tonic vibration reflex is a polysynaptic spinal cord reflex involving Ia spindle afferents. Interestingly, in patients with writer’s cramp vibration of the dystonic limb at rest induces involuntary dystonic co-contractions of the involved muscles (Kaji et al., 1995a), which is relieved by blocking Ia muscle afferents with intramuscular injection of lidocaine (Kaji et al., 1995b). In addition, Grünewald et al. (1997) showed that perception and motion of the tonic vibration reflex is abnormal, but not of position, suggesting that an impaired perception of movement. Neurophysiologic approaches employing somatosensory evoked potentials (SSEPs) suggest that patients with focal dystonia have an abnormal processing of somatosensory information within the lemniscal system [for a review see Tinazzi et al. (2003)]. Indeed, the recovery function of the SSEP after paired median nerve stimulation showed an impaired inhibition at the spinal and cortical level (Frasson et al., 2001).

In line with these findings, Abbruzzese et al. (2001) reported defective suppression of the motor evoked potential (MEP) caused by peripheral electric stimulation in patients with FHD. Overall, these studies conclude that dystonic patients may have difficulty interpreting sensory input that occurs before, and, in more severely affected patients, even during movement (Murase et al., 2000).

## Repetitive Movements and Abnormal Sensory-Motor Plasticity

Several evidences suggest that focal dystonia can be triggered by periods of intensive training and repetitive movements. Byl et al., 1996; Byl and Melnick, 1997) trained primates in a repetitive, intensive motor task over 12–25 weeks producing a significant distortion of the hand sensory representation culminating in motor symptoms resembling those seen in FHD. Similar results are obtained in monkeys after surgical joining of the skin of adjacent digits, which increases sensory drive to motor cortex (Clark et al., 1988). An increased sensory drive can also be obtained by using a protocol of associative (Hebbian) pairing of tactile stimulation (APTS). Reversible reorganization of the adult rat paw representations in somatosensory cortex (SI) can be induced by a few hours of APTS with a selective enlargement of the areas of cortical neurons representing the stimulated skin field. This plastic changes are probably engaging glutamatergic synapses (Godde et al., 1996). Using an analogous APTS protocol in human beings revealed an increase of spatial discrimination performance indicating that fast plastic processes based on co-activation patterns act on a cortical and perceptual level. Also, synchronous stimulation of peripheral muscles induces organizational changes in motor representations, characterized by an increase in motor map size of stimulated muscles and a reduction in map separation, as assessed using transcranial magnetic stimulation (TMS) (Schabrun and Ridding, 2007). Similar abnormalities of cortical organization are seen in both motor (Byrnes et al., 1998) and sensory cortices (Meunier et al., 2001; Butterworth et al., 2003) representations in FHD. Probably, the most important neurophysiological correlates of focal dystonia are the enlarged and partially overlapping receptive fields in the somatosensory and motor cortices of patients with writer’s cramp (Meunier et al., 2001). This “smearing” of receptive fields mimic those seen in primate models of FHD [see below, Byl et al. (1996)]. It is believed that the lack of clearly defined somatosensory and motor cortical representations leads to the involuntary motor output seen in dystonia. This altered somatosensory somatotopy can be observed also at subcortical level. Patients affected with severe generalized dystonia when undergoing presurgical microelectrode exploration of basal ganglia exhibit a loss of selectivity of somatosensory neurons in the internal part of globus pallidus (Vitek et al., 1999) and in the sensory (ventral caudal) and cerebellar (ventral intermediate) relay nuclei of the thalamus (Lenz et al., 1999). Indeed, receptive fields are unusually large, and an abnormally high proportion of them include multiple parts of the body. The basal ganglia have been attributed a role in “sensory gating,” filtering out what sensory information is conveyed to the motor system (Murase et al., 2000). To this respect, an important role is played by cholinergic interneurons. Although their firing activity appears to be unrelated to movements they discharge phasically in response to sensory stimuli serving as a cue for reward delivery and consumption (Graybiel et al., 1994). Moreover, recent work reveals that thalamic projections to the striatum engage cholinergic interneurons to modulate corticostriatal inputs, thereby supporting their fundamental role in filtering excitatory afferences (Ding et al., 2010). Several evidences in animal models suggest that the presence of an enhanced acetylcholine tone in the striatum of mutant human torsinA mice, as compared with both normal human torsinA and NT littermates (Pisani et al., 2006). The role of cholinergic transmission cannot be neglected in dystonia considering that anticholinergic drugs are the only (partially effective) available treatment in dystonia (Fahn, 1983). Also, cerebellum exerts a powerful influence over the somatosensory system especially after repetitive somatosensory stimulation (Ben Taib et al., 2005). The effect of cerebellum on the somatosensory system is very powerful since it may tune out the somatosensory threshold in the cortex playing a role in both temporal and spatial discrimination (Pastor et al., 2004). Numerous studies have demonstrated that the cerebellum is involved in sensorimotor adaptation (Popa et al., 2013; Sadnicka et al., 2014), and cerebellar dysfunction in dystonia might therefore affect sensorimotor adaptation. In keeping with these findings, eye blink conditioning is altered in patients with various forms of focal dystonia (Teo et al., 2009), and saccadic adaptation is impaired in patients with myoclonus-dystonia (Hubsch et al., 2013).

Taken together, these data suggest that overtraining itself even in normal subjects may induce profound changes in connectivity in the sensory and motor cortices but they do not give clues why in human beings only some subjects develop dystonia after excessive training whereas others are completely healthy. It can be postulated that the abnormal sensorimotor representations seen in FHD are due to an abnormally increased response to repeated patterns of stereotypical and convergent afferent inputs. This hypothesis receives some support by the finding of abnormally increased representational plasticity in FHD (Quartarone et al., 2003). It is likely that subtle abnormalities of plasticity may render some individuals susceptible to dystonia if plastic changes are pushed to their extreme by frequent repetition (Quartarone et al., 2006). According to the two factor hypothesis dystonia is triggered and maintained by the concurrence of environmental factors such as repetitive training and abnormal mechanisms of plasticity within somatosensory loop (Quartarone et al., 2006; Quartarone and Hallett, 2013). There is considerable evidence suggesting that both the motor and sensory cortex in primary dystonia exhibits an exaggerated responsiveness to TMS conditioning protocols. One of these protocols is paired associative stimulation (PAS) where median nerve stimulation is coupled with TMS delivered over the motor are of the opposite hemisphere (Stefan et al., 2000). Patients with writer’s cramp showed a larger increase in excitability of the targeted corticospinal output neurons after PAS. An important feature of PAS-induced associative plasticity in healthy controls is input specificity as PAS after effects are largely confined to the cortical target representation receiving a dual congruent input. Hence, the increase of corticospinal excitability is significant in abducot pollicis brevis (APB) muscle, which in innervated by median nerve, with no effects on surround muscles such as abductor digiti minimi (ADM). This spatial specificity was lost in WC patients were PAS induced an increase in corticospinal excitability in median (APB) and ulnar (ADM) innervated muscles (Quartarone et al., 2003). This loss of spatial specificity appears to be a relevant finding and could be related to the abnormalities of neuronal inhibition previously identified both in the motor and somatosensory system in dystonic patients (Berardelli et al., 1998). Since, Stefan et al. (2000) have shown that plastic changes occur at the level of the motor cortex, our findings indicate an increased modifiability of the motor cortex in patients with writer’s cramp to reorganization driven by sensory input from the affected hand.

This hallmark of aberrant plasticity is not confined but tends to generalize across the entire sensory-motor loop representing the most important endophenotypic trait of dystonia (Quartarone and Hallett, 2013).

## Non-Invasive Neuromodulation as Therapeutic Approach in FHD

Treating FHD is challenging and most of the times botulinum toxin, which is the gold standard, lacks of efficacy. Therefore, alternative forms of treatment are urgently needed. Hence, the identification of the putative mechanisms underlying dystonia may help the design of novel therapeutic strategies based upon physiological findings. In the present section, we will focus on treatments using NIBS. Considering the loss of inhibition is one of the most important hallmarks in the pathophysiology of dystonia, then augmenting inhibition may be an useful strategy to relieve dystonic postures. In line with this hypothesis, it has been reported that 1 Hz repetitive transcranial stimulation (rTMS) over primary motor cortex may normalize intracortical inhibition and induce a mild improvement in motor performance (Siebner et al., 1999). Another potential target is premotor cortex (PMC), which exerts a powerful control on sensory-motor integration. Huang et al. (2004) demonstrated that 1 Hz rTMS may normalize the reciprocal inhibition in writer’s cramp and improve the rating of handwriting in which lasted up to a few hours in most patients (Murase et al., 2005). These findings are in line with open data on bilateral epidural PMC stimulation showing a significant improvement after at least 1 month of continuous stimulation (Lalli et al., 2012). The efficacy of rTMS over the PMC in all of these studies is not surprising considering the strategic role of PMC in sensory-motor integration and motor learning. Two more studies have used rTMS a potential treatment in FHD; however, the lack of sham stimulation has made data interpretation very difficult. Lefaucheur et al. (2004) administered daily sessions of inhibitory rTMS over the PMC for five consecutive days in three patients with severe generalized secondary dystonia and they found a significant reduction in the severity of the Burke–Fahn–Marsden scale. The same protocol in a patient with segmental dystonia involving the neck and the right arm induced a moderate improvement in symptoms and function relating to amelioration in the neck dystonia for 4 months after the stimulation; no changes were noted in the right arm dystonia (Allam et al., 2007). Another potential NIBS approach is transcranial direct current stimulation (tDCS). tDCS was tested in a randomized double-blind sham-controlled study. Benninger et al. (2011) investigated the efficacy of cathodal stimulation in patients with writer’s cramp and they found no favorable effects on clinical scales and failed to restore normal handwriting kinematics and cortical inhibition. Finally, considering that afferent input is known to be a powerful driver of cortical reorganization, it has been suggested that one strategy to re-establish discrete cortical representations and alleviate dystonic symptoms may be to provide independent input from involved muscles through asynchronous afferent stimulation in which there is no consistent temporal coupling of the evoked afferent inputs. This is in keeping by the finding that reducing correlated input from adjacent digits, by surgical separation of syndactyly, produces separation of digital cortical representations (Mogilner et al., 1993). Therefore, it seems feasible that asynchronous, and non-associative, stimulation (NAS) of hand muscles may temporarily reverse representational changes characteristic of FHD. NAS was applied in a population of patients affected by writer’s cramp. Prior to NAS, dystonics had larger smeared maps with the centers of gravity (CoGs) of the first dorsal interosseus (FDI) and abductor pollicis brevis (APB) maps were closer together. Dystonics had impairments in grip-lift, handwriting, and cyclic drawing tasks. After NAS, map size was transiently reduced in all muscles in dystonic participants and FDI and APB CoGs moved further apart. In addition, NAS produced a transient reduction in movement variability during cyclic drawing (Schabrun et al., 2009). The mechanism of action of NAS is unknown, however, since cortical representations are maintained through inhibitory GAbaergic intracortical circuits (Sanes et al., 1998), it is likely that NAS may act by renfocing this inhibitory interneruons that are impaired in FHD (Schabrun et al., 2009).

## Conclusion

Despite NIBS represents a promising therapeutic tool in dystonia, the overall improvements so far have not been sustained in all studies. This is not surprising since as we discussed in the previous sections enhanced plasticity causes inappropriate association between sensorimotor inputs and outputs. In this case, a short session of NIBS might have little immediate effect on dystonic movements, because bad motor memories have already been “learned,” and takes time to be erased (Quartarone and Hallett, 2013). It is likely that, as deep brain stimulation, longer NIBS sessions are needed to reverse a process related to an abnormal sensorimotor plasticity that developed over several years. In this context, rTMS could be used in the near future better to select dystonic patients as good responders for an eventual epidural PMC implantation. Finally, NIBS, such as NAS, should be extensively used in the near future to empower rehabilitation treatments.

## Conflict of Interest Statement

The authors declare that the research was conducted in the absence of any commercial or financial relationships that could be construed as a potential conflict of interest.
